# Silicon-mediated drought resilience mechanisms in crops: from physiology to molecular insights

**DOI:** 10.3389/fpls.2026.1788106

**Published:** 2026-04-28

**Authors:** Olajumoke Ogedengbe, James Hunt, Alexis Pang, Dorin Gupta

**Affiliations:** School of Agriculture, Food and Ecosystem Sciences (SAFES), Faculty of Science, The University of Melbourne, Melbourne, VIC, Australia

**Keywords:** abiotic stress, crops, drought, resilience, silicon, stress mitigation

## Abstract

Drought stress increasingly constrains crop growth, yield, and quality through reduced photosynthesis, impaired nutrient uptake, and oxidative damage, with recent occurrences causing substantial yield losses across major production regions. Silicon (Si), although not an essential nutrient, is a prevalent, non-toxic, “quasi-essential” element that can enhance crop performance under water limitation; however, Si-induced drought-mitigation mechanisms are frequently reported in isolation, based on individual studies. This review aims to provide a holistic understanding of Si’s role in enhancing plant resilience to water stress by comprehensively compiling and integrating physiological to molecular mechanisms underpinning Si-mediated drought resilience, while contextualising soil Si availability, plant uptake and accumulation diversity, and agronomic delivery options. Across crops, Si fertilisation consistently improves water relations by promoting root growth, hydraulic conductance, and aquaporin regulation, enabling osmotic adjustment and modulating stomatal behaviour through hormone-reactive oxygen species interactions. Evidence is synthesised from controlled and field studies, alongside temporal trend analyses showing a shift from uptake/transport research (pre-2010) to physiological characterisation (2010–2015), and to integrative multi-omics (2020–present), including transcriptomics and metabolomics. Key research gaps include limited field validation, inconsistent experimental designs and drought imposition protocols, incomplete multi-omics-to-trait integration, and underexplored Si-microbiome interactions. Optimising the Si source, dosage, timing, and application method across different species and environments presents a practical approach to developing climate-resilient, low-input cropping systems in the face of increasing drought risk.

## 1 Introduction

Drought stress is a significant environmental challenge that adversely impacts plant growth, development, and productivity. It limits crop yield and quality, particularly in semi-arid and arid dryland regions (Seleiman et al., 2021). Drought stress induces physiological, morphological and molecular changes which impacts plant processes (Ilyas et al., 2021). The primary effect of drought stress in plants is a reduction in photosynthesis, primarily attributed to the inhibition of CO_2_ assimilation due to stomatal closure (Wang et al., 2018). In some cases, it is also attributed to reduced radiation interception due to inhibited leaf area growth (Earl and Davis, 2003). Additionally, the capacity for light absorption is diminished because of a decrease in chlorophyll content, resulting from increased chlorophyll degradation, inhibited chlorophyll synthesis, and damage to chloroplast structures Moreover, plants experience disruptions in nutrient uptake during drought stress, which impedes their growth and interferes with critical reproductive processes, ultimately leading to low yields and poor-quality produce (Maghsoudi et al., 2016a). Under water stress, plants activate drought response mechanisms to cope with the limited water supply. These mechanisms include osmotic regulation, activation of the antioxidant defence system, protection of the photosynthetic apparatus and related organelles, and stomatal closure to prevent water loss through transpiration (Fang and Xiong, 2015; Seleiman et al., 2021). However, these response mechanisms also alter other plant processes. For instance, the closure of stomata to prevent water loss results in reduced CO_2_ assimilation, impairing photosynthesis. Ultimately, reduced plant growth, water uptake, and root exploration disrupt nutrient uptake during periods of drought stress, further impeding overall plant growth (Wang et al., 2018).

In recent years, arid and semi-arid grain-producing regions globally have experienced recurrent droughts (Wing et al., 2021). In Australia, the drought of 2019–2020 resulted in a significant decline in crop yields, particularly for wheat, barley, and canola, leading to a 10% reduction in national wheat output, with some regions experiencing losses of up to 50% (ABARES, 2020). Similarly, the 2020–2021 agricultural season witnessed substantial crop losses attributable to drought, bushfires, and floods. Wheat production was notably affected, with an estimated 11% decrease in yield compared to preceding years (ABS, 2021). On a global scale, drought has had a similar negative impact on crop production. In the United States, a drought in 2021 resulted in a significant decline in yields of maize and soybeans, with a reported 4% reduction in maize production (United States Department of Agriculture (USDA), 2021). In Europe, the drought of 2022 caused crop yield reductions of up to 30% for major crops such as maize and wheat, particularly in southern and eastern regions (European Commission, 2022). These trends underscore the vulnerability of agricultural systems to climate variability, highlighting the necessity for adaptive strategies to safeguard food security. Given the continuously evolving global climate, plants will be required to adapt to an expanding array of environmental conditions in the future (Mishra and Singh, 2010; Swann, 2018). The anticipated increase in future drought occurrence due to the increasing impact of climate change is a major threat to global food and nutrition security (Lee et al., 2023). Consequently, to boost production and fulfil the rising global food demand, it is imperative to systematically enhance plants’ resilience to drought stress using sustainable methods (Trenberth et al., 2014; Wing et al., 2021).

**Figure 1:**
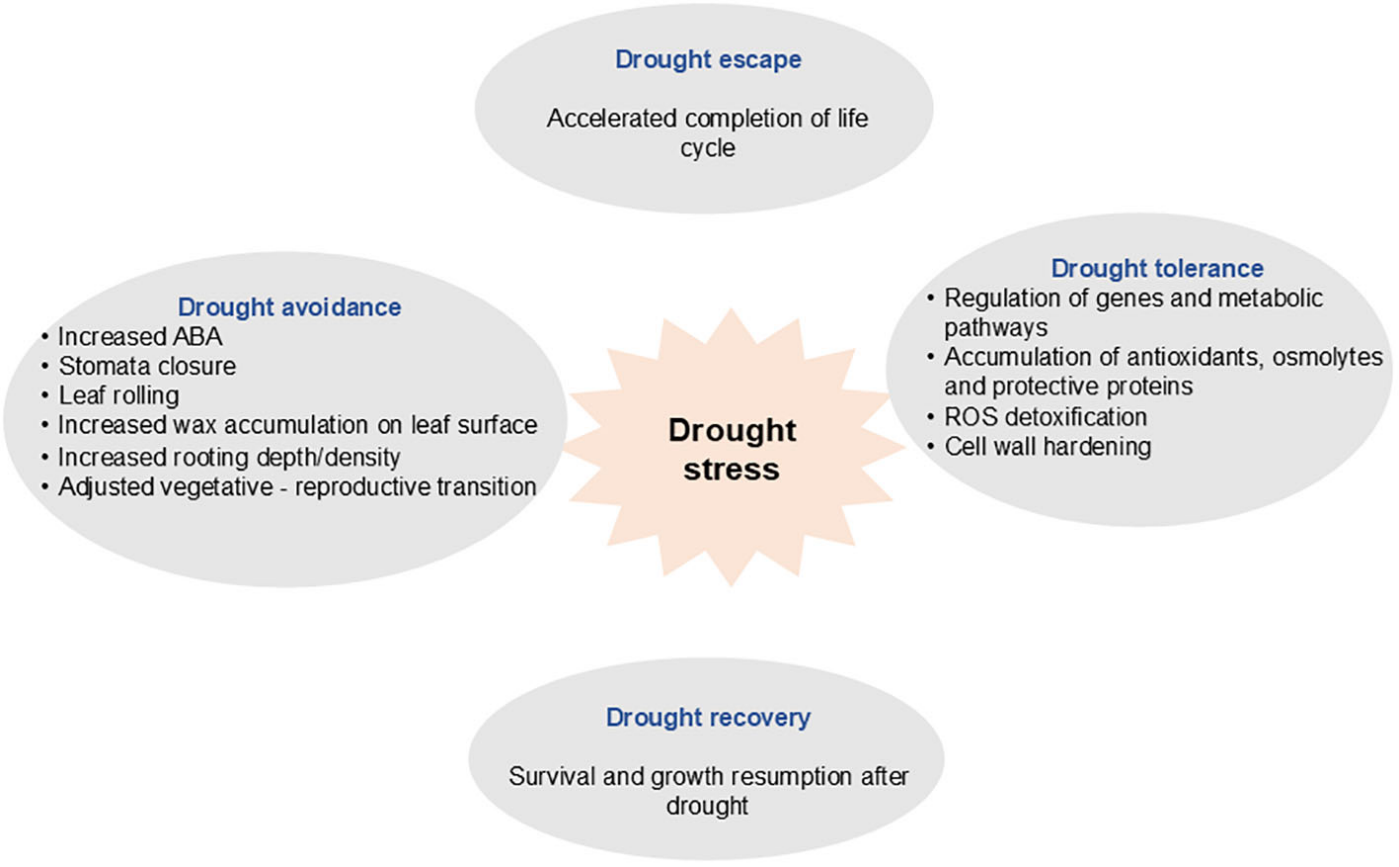
Key strategies employed by plants to cope with drought stress.

Plants have evolved range of strategies to sustain reproductive output under conditions of drought stress. This includes drought avoidance, drought tolerance, drought escape, and drought recovery (Fang and Xiong, 2015) (**Figure 1**). Drought avoidance involves mechanisms that reduce water loss by regulating transpiration through stomatal control, while concurrently maintaining water uptake via an extensive root system (Farooq et al., 2009). Drought tolerance entails adjustments to sustain physiological and biochemical processes within plants whilst under water deficit. Drought escape involves accelerated development and a shortened life cycle to evade the detrimental effects of water stress during critical reproductive stages (Shavrukov et al., 2017). Drought recovery refers to the capacity of plants to survive and resume growth and development following exposure to drought stress (Vilonen et al., 2022).

Drought avoidance and tolerance strategies are critical factors that enhance plant resilience. Key mechanisms include improving water use efficiency through partial stomatal closure and increasing the root-to-shoot ratio, activating antioxidant defence mechanisms, undergoing osmotic adjustments, and elevating phytohormone synthesis (Fang and Xiong, 2015; Seleiman et al., 2021). Plants undergo a variety of physiological, biochemical, morpho-anatomical, and molecular changes in response to drought stress. These adaptations facilitate the plant’s ability to manage conditions of limited water availability (Kalefetoğlu and Ekmekci, 2005). However, under conditions of severe or prolonged drought, these stress response strategies may prove inadequate for maintaining plant growth, as the level of ROS exceeds the capacity of antioxidants, leading to an imbalance in oxidative homeostasis (Kalefetoğlu and Ekmekci, 2005). Consequently, enhancing plant resilience to drought stress, either through genetic improvement or the exogenous application of supportive elemental products to bolster drought response strategies, is crucial for achieving increased productivity.

Silicon plays a crucial role in enhancing plants’ ability to cope with abiotic and biotic stressors. Under drought conditions, Si enhances plant drought tolerance by maintaining water relations through improved root water uptake and retention. Sustaining photosynthetic activities by preserving chlorophyll content and photosystem II efficiency, and it protects cellular organelles from oxidative stress by increasing antioxidant enzyme activity thereby stabilising cellular structures (Saja-Garbarz et al., 2024). It also engages in intricate signalling pathways that involve close interactions with key molecules, including hormones such as jasmonic acid (JA) and abscisic acid (ABA), as well as reactive oxygen species (ROS) within plant cells (Tripathi et al., 2021a). Analysis of 107 articles investigating recent advancements in the effects of Si application on crops under drought conditions substantiated that soil Si positively influences the growth and development of major crops, particularly under environmental Stress (Rea et al., 2022). Similarly, Wang et al. (2021b) reviewed 137 studies on the impact of Si on drought stress responses across various plant species. They concluded that further research is necessary to explore the distribution and drought mitigation strategies of Si under stress conditions, particularly in crops with differing Si accumulation capacities.

Recent studies demonstrating improved drought stress tolerance in plants include those by (Abdullah et al., 2025; Biju et al., 2023a; El-Okkiah et al., 2022; Saja-Garbarz et al., 2024). Research on other stressors encompasses studies on salinity (Shen et al., 2022; Stadnik et al., 2022), heat (Ashfaq et al., 2022), UV-B radiation (Mavrič Čermelj et al., 2022), acid rain (Ju et al., 2017), metal toxicity (Wang et al., 2020; Rahman et al., 2021); fungal pathogens (Li et al., 2019; Lin et al., 2020) and insect attack (Hall et al., 2020; Islam et al., 2023). While most studies focus on isolated mechanisms through which Si mitigates stress in plants, this article addresses the research gap by comprehensively synthesising the mechanisms underlying Si-mediated drought tolerance in plants. It explores various aspects of Si -mediated drought tolerance, including physiological and biochemical processes, morpho-anatomical changes, and molecular mechanisms. By summarising the intricate interactions between Si supplementation and plant responses to drought stress, the article offers insights into the potential of Si as a sustainable approach to improving crop productivity under water-limited conditions. Additionally, the review discusses Si availability in soil, uptake by plants, and variation in Si accumulation among plant species. Overall, it aims to provide an integrated perspective on the potential role of Si in enhancing drought tolerance in plants through various interconnected mechanisms. This knowledge is intended to inform strategies aimed at enhancing crop resilience to water stress, thereby contributing to the development of resilient and sustainable cropping systems capable of withstanding the increasing challenges posed by climate change.

## 2 Research trends and emerging hotspots in Si-mediated drought tolerance

### 2.1 Evolution of research on Si in drought stress

**Figure 2:**
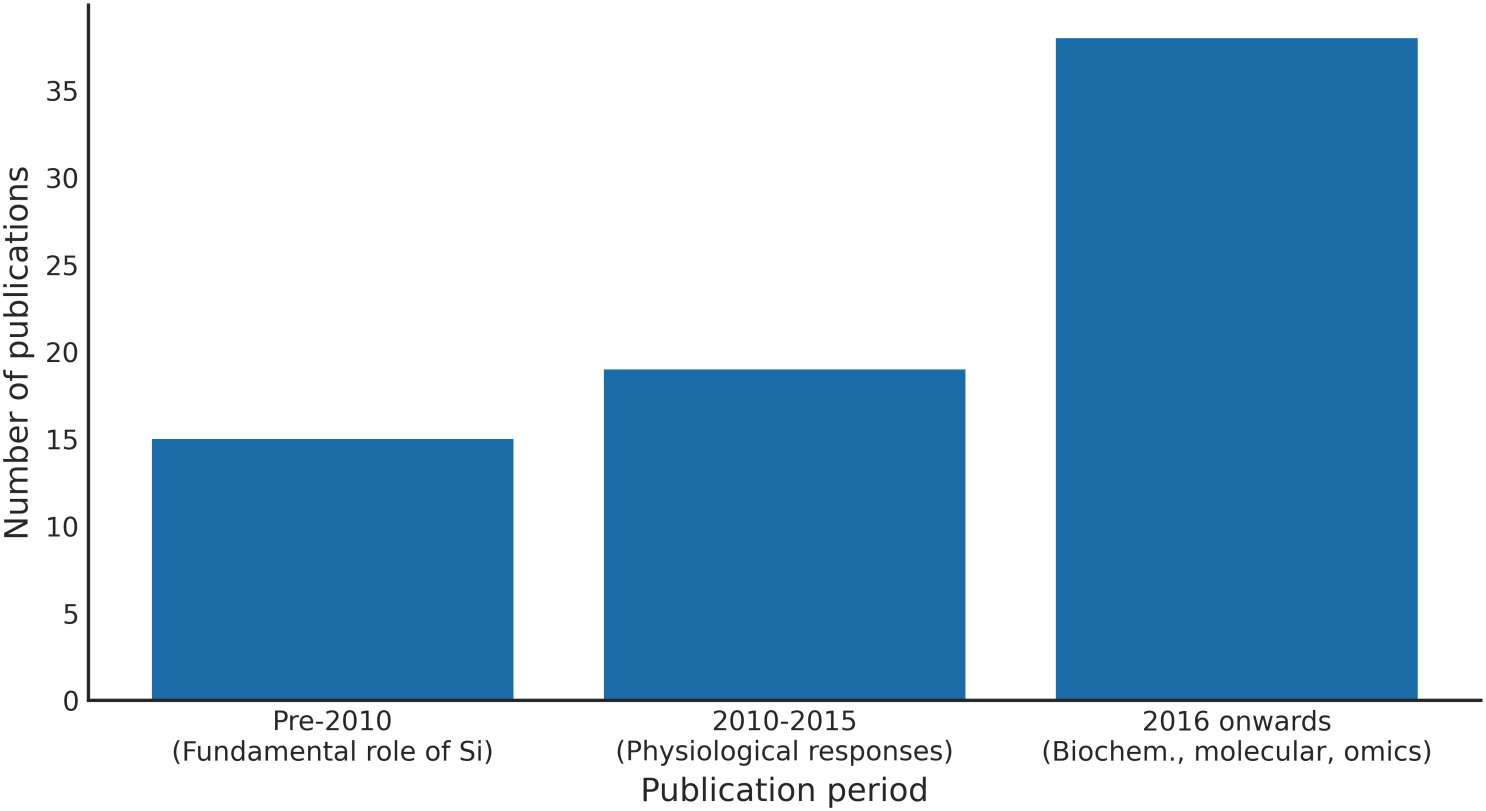
Trends in Si-related research publications included in this review.

Research on Si in drought tolerance has evolved from foundational studies on its uptake and transport (Chiba et al., 2009; Deshmukh et al., 2013) to more intricate investigations into stress physiology and molecular regulation. Early studies (pre-2010) primarily established Si’s fundamental role in plant biology, concentrating on its uptake mechanisms, transport systems, and classification as a beneficial element. These foundational studies provided a basis for understanding Si accumulation and its variability across different species. Between 2010 and 2015, the focus of research shifted towards physiological responses, with studies demonstrating Si’s capacity to enhance photosynthetic performance, regulate stomatal conductance, and improve plant water status under drought conditions. This period marked a transition from descriptive observations to the functional characterisation of Si-mediated stress mitigation (Ahmed et al., 2011; Chen et al., 2011). From 2016 onwards, research expanded significantly, incorporating biochemical and molecular perspectives (Ma et al., 2016; Biju et al., 2017). This period saw a notable acceleration in research output, reflecting increased global interest in sustainable approaches to managing abiotic stress. Recent studies (2020–present) indicate a clear shift towards integrative approaches, including transcriptomics and metabolomics (Jiang et al., 2022; Ning et al., 2026; Devkar et al., 2025). **Figure 2** illustrates the trends and growth in publications on Si and drought stress included in this review, highlighting the significant increase in publications from 2015, which correlates with the recent surge in Si research focused on stress tolerance. The temporal and thematic analysis of published literature included in this review is provided in the **Supplementary Article**.

### 2.2 Emerging research frontiers

Recent research has increasingly focused on the application of high-throughput molecular techniques to elucidate the regulatory networks underpinning Si-mediated drought tolerance. Transcriptomic and metabolomic analyses have identified differentially expressed genes and metabolic pathways associated with stress responses, including those linked to antioxidant activity, hormone signalling, and transport processes (Biju et al., 2023b; Jiang et al., 2022; Ning et al., 2026). Particular emphasis has been placed on the interaction between Si and phytohormones, especially abscisic acid (ABA), which plays a crucial role in drought signalling (Gao et al., 2022). The integration of multi-omics datasets is emerging as a robust approach for correlating molecular alterations with physiological outcomes. This systems-level perspective is anticipated to provide deeper insights into the complex regulatory networks that govern plant responses to drought stress.

### 2.3 Synthesis and research gaps

Despite significant advances, several critical gaps remain in the current body of research. First, most studies have been conducted under controlled conditions, with limited validation in field environments. Bridging this gap is essential for translating laboratory findings into practical agricultural applications. Second, there is a need for greater integration of multi-omics data with phenotypic and agronomic traits. While molecular studies have identified numerous candidate genes and pathways, their functional validation and relevance to crop performance remain limited. Third, inconsistencies in experimental design, including variations in Si sources, application methods, and concentrations, hinder effective comparisons across studies. Standardising methodologies would improve reproducibility and facilitate meta-analyses. Finally, the potential role of Si in modulating plant-microbiome interactions under drought stress remains underexplored and represents a promising avenue for future research. Addressing these gaps will be critical for translating research into practical agricultural applications.

## 3 The importance of Si in sustainable agriculture

Silicon is prevalent in soil and is considered environmentally friendly due to its non-toxic properties. This characteristic renders it an essential element for sustainable agriculture and aligns effectively with the principles of organic production (Verma et al., 2021a; Tayade et al., 2022). Silicon fertilisation enhances tolerance to drought, salinity, and diseases, resulting in higher and more stable yields that may increase by 10-50%, thereby improving farm profitability and resilience to climatic variability (Artyszak, 2018; Wang et al., 2021a). Silicon strengthens both physical and biochemical defences to such an extent that the use of fungicides and pesticides can be reduced without compromising yield. For instance, in rice cultivation, Si alone has been demonstrated to control blast as effectively as fungicides while increasing yield more than fungicide applications, thus facilitating a reduction in chemical inputs and the associated environmental impacts (Seebold et al., 2004). This decrease in agrochemical inputs, coupled with improved water retention and nutrient use efficiency in Si-amended soils, promotes cleaner water cycles, enhances water reusability, lowers environmental footprints, and fosters more climate-resilient production systems (Kovács et al., 2022; Parizad and Bera, 2023). By improving yield stability and crop health under stress conditions of heat, drought, and pest pressures, Si helps optimise yield potential in stressful environments. This reduces the need to expand cultivated land. Additionally, it can support farmers’ livelihoods in areas that are becoming more vulnerable to climate extremes, thus alleviating societal pressures related to food and feed security. This multifunctionality positions Si application as a promising tool for climate-smart, low-input cropping systems that aligns with the global transition towards sustainable food production.

### 3.1 Silicon’s prevalence in earth’s crust and its impact on crop stability

Silicon constitutes 27.7% of earth’s crust, making it the second most abundant element after oxygen, which accounts for 46.6% (Devanna et al., 2021). Although the occurrence of pure Si is rare, it predominantly exists in nature as silica (SiO_2_) or silicates due to its strong affinity for oxygen. Approximately 60% of the earth’s crust is composed of SiO_2_, and silicate minerals constitute 90% of the mass of the earth’s crust (Ma and Takahashi, 2002; Mitra, 2015). In the 1800s, botanists discovered that plants possess higher concentrations of Si compared to other mineral elements. Although the scientific community did not regard Si as essential for plant development, some early researchers suggested that Si might enhance the rigidity of cereal stalks, thereby mitigating lodging in plants (Datnoff and Rodrigues, 2015). Increased vulnerability to lodging has been observed in wheat and rice when there is a deficiency of Si (Liang et al., 1994; Savant et al., 1996). Epstein (1999) reported that a substantial body of research supports the conclusion that Si enhances plant growth and development with effects observed directly attributed to the element, which has led to its classification as a “quasi-essential” element. In the early 20th century, Si was acknowledged as one of the 15 elements necessary for plant development (Tubana et al., 2016). Recently, it has been attracting increasing global attention, particularly due to the growing impact of climate change on food production.

The Si content in soil ranges from 23% to 35%, with primary and secondary silicate minerals being the principal sources (Mitra, 2015). However, a high Si content in soil does not necessarily equate to high Si availability for plants, as Si is typically present in various forms within the soil, predominantly as insoluble oxides and silicates, which are not accessible for plant uptake (Hodson et al., 2005). Plants can only absorb Si in the form of monosilicic acid (H_4_SiO_4_) (Epstein, 1994), referred to as plant available silicon (PAS). The concentration of plant−available silicic acid in soils typically ranges from 0.1–0.6mM (≈10 to >100 mg kg^-^¹), and PAS values below 20mg kg^-^¹ in soils are indicative of Si deficiency (Epstein, 1994; Liang et al., 2015). These thresholds underscore the agronomic rationale for targeted Si supplementation in many cropping systems.

### 3.2 Influence of Si on soil microbial activity

Silicon application significantly influences soil microbial activity by enhancing community resilience, diversity, and function under various stresses. In wheat, Si stimulated beneficial bacteria such as *Acidobacteria* and *Thaumarchaeota*, which significantly mitigated cadmium toxicity (Song et al., 2021). By decreasing the bioavailability of metals and metalloids and buffering abiotic stress, Si alleviates toxicity constraints on beneficial taxa and favours microbial communities involved in nutrient cycling and detoxification (Deng et al., 2021; Song et al., 2021; Etesami, 2024). In sugarcane, Si enhanced the abundance of *Proteobacteria* and enriched the rhizosphere bacterial network, which improved plant growth and productivity (Deng et al., 2021). Nanosilica increased soil enzyme activities, the relative abundance of beneficial microorganisms, and improved overall microbial diversity and operational taxonomic units, thereby facilitating mineralisation and the release of nutrients (Zhu et al., 2024; Etesami, 2024; Shi et al., 2025). These effects are influenced by the modification of plant water relations, nutrition, and defence signalling by Si, which in turn alters both the quantity and composition of root exudates that feed and select rhizosphere bacteria (Deng et al., 2021; Ahmad et al., 2024b). Across multiple systems, Si fertilisation alters rhizosphere pH, available Si, P and K, organic C, and N forms, and these soil variables emerge as primary drivers of bacterial community shifts in ordination and network analyses (Deng et al., 2021; Mallano et al., 2022; Etesami, 2024; Gao et al., 2025). Reviews on Si and Si nanoparticles emphasise that Si sources, application rates, baseline pH, pathogen status, and background soil properties determine whether Si primarily stimulates beneficial bacteria, shifts bacteria:fungi ratios, or modulates pathogen-suppressive functions in a given soil–crop system (Rajput et al., 2021; Verma et al., 2022a; Etesami, 2024). While these studies focus on stresses such as diseases, salinity, heavy metals, and low temperature, there remains a lack of research addressing the role of Si in modulating plant-microbiome interactions under drought stress.

### 3.3 Silicon fertiliser sources, forms, and application methods for crop stress mitigation

Silicon fertilisers are available in both solid and liquid forms, derived from a range of sources with distinct solubility and agronomic behaviour. Solid minerals and by-product sources, such as calcium silicate slags, are most effective in acidic to neutral soils, where dissolution occurs more rapidly. Natural silicate minerals and biogenic silica, such as wollastonite, exhibit slower dissolution rates, while diatomaceous earth provides more soluble amorphous Si. Soluble silicates, including sodium or potassium silicates and stabilised monosilicic acid, offer highly soluble and rapidly released Si (Ning et al., 2014; Zellner et al., 2021). Silicon can be supplied via soil, fertigation, root application, foliar application, or seed priming, depending on the source. Soil application using granular Si, such as calcium silicate or amorphous silica, is the most widely used method in field crops. This approach is particularly significant for long-term stress mitigation, as it establishes a pool of plant-available monosilicic acid in the rhizosphere (Johnson et al., 2022; Madegwa et al., 2025). Soluble Si can be applied through fertigation, root application in hydroponics, or foliar application (dos Santos Sarah et al., 2021; Tebow et al., 2021; Saja-Garbarz et al., 2024). In fertigation, Si is introduced through the irrigation system, while in hydroponics, it is added directly to the nutrient solution. Foliar application facilitates rapid and flexible interventions. Seed priming is a suitable method for early-stage stress mitigation; it involves soaking seeds in a Si solution, such as sodium or potassium silicate or nano-silica, prior to sowing. This technique has been shown to enhance germination percentage and seed vigour under polyethylene glycol-induced drought stress (Biju et al., 2017). Nanosilica (silica nanoparticles, SiNPs) is gaining increasing recognition as a source of Si for plants, attributed to its superior bioavailability, translocation, and reactivity compared to other Si forms. This characteristic renders SiNPs particularly effective in enhancing drought tolerance in plants (Rakesh et al., 2024). When administered via foliar spray, soil incorporation, or seed priming, SiNPs facilitate an efficient increase in Si accumulation within plant tissues, mitigate oxidative damage induced by drought, and promote enhanced growth and yield (Raza et al., 2023; Sulaiman et al., 2023). The various delivery options allow for fine-tuning of Si nutrition, enabling growers to align the Si source, dosage, and application method with crop type, soil Si status, and the predominant stress regime, thereby maximising both short-term protection and long-term resilience.

### 3.4 Silicon uptake mechanisms and accumulation in crops

The concentration of Si in plants ranges from 0.1% to 10% dry weight (DW) or higher, depending on the plant’s uptake capacity (Ma and Yamaji, 2006). The accumulation of Si is influenced by the plant’s uptake mechanism. Active uptake results in high Si accumulation, while Si accumulation in plants with passive uptake is lower than those with active uptake, as it is constrained by transpiration and water flow rates. Rejective uptake leads to low Si content in plant tissues, even when the external solution contains adequate PAS levels. **Table 1** presents a summary of these mechanisms. Dicots, such as those in the Fabaceae and Solanaceae families, are characterised as Si excluders (low Si accumulators) due to their rejective uptake mechanism, resulting in the absorption of less than 1% DW Si in their tissues (Ma and Takahashi, 2002). In contrast, monocots are high accumulators, facilitated by an active uptake mechanism, with the Poaceae family accumulating between 5% and 10% DW Si (Ma and Takahashi, 2002). Certain dicots within the Cucurbitaceae family are intermediate accumulators, employing a passive uptake mechanism and accumulating between 1% and 3% DW Si (Gunes et al., 2008; Kaur and Greger, 2019).

**Table 1:** Summary of Si uptake mechanisms and accumulation in plants.

Uptake mode	Mechanism	Typical plants	Relative Si in plant	Effect on soil solution	Transporters	References
Active	Energy-dependent, faster than water uptake	Rice (*Oryza sativa)*, wheat (*Triticum aestivum*), maize (*Zea mays*)	High	Si decreases sharply	Lsi1, Lsi2 (highly expressed)	(Mitani and Ma, 2005)
Passive	Diffusion/mass flow, aligns with water uptake	Cucumber (*Cucumis sativus*), oat (*Avena sativa)*	Medium	Si stable	Transporters low/absent	(Faisal et al., 2012)
Rejective	Exclusion/slower than water uptake	Tomato (*Solanum lycopersicum)*, legumes	Low	Si more concentrated than in plants	Transporters low/absent	(Mitani and Ma, 2005)

It is important to note that Si cannot be assimilated at elevated concentrations because silicic acid begins to polymerise at high concentrations, thereby reducing its mobility and uptake efficiency (Tamai and Ma, 2003). Therefore, to optimise Si nutrition and fully realise its stress−mitigating benefits, Si must be supplied at concentrations that maintain silicic acid predominantly in its monomeric form while matching plant demand and soil buffering capacity.

Studies suggest that variation in Si absorption and mobilisation among different plant species may have significant implications for the drought-mitigating effects of Si on plants (Ma and Takahashi, 2002) suggested that the beneficial impact of Si is associated with its accumulation in plant shoots, implying that Si accumulators derive greater benefits from Si. Conversely (Katz, 2014) contended that the advantages of Si are not confined to Si accumulators, as low Si accumulation does not necessarily equate to low Si functionality in plants. Wang et al. (2021a) asserted that while both groups of plants benefit from Si through physical barriers and biochemical processes, high Si accumulators derive greater advantages from Si due to the development of mechanically reinforced tissues and the establishment of physical barriers. In contrast, the benefits for low Si accumulators are predominantly realised through biochemical pathways. Given that Si has been demonstrated to effectively mitigate both abiotic and biotic stresses in both groups, detailed comparative studies are now required to disentangle the specific processes associated with accumulators versus excluders under drought and other stress conditions, and to optimise these processes for crop improvement and management.

### 3.5 Alleviation of abiotic and biotic stresses

Silicon reinforces defence mechanisms, thereby enhancing plant resilience against both abiotic and biotic stresses (Currie and Perry, 2007). The stress mitigating effects of Si are mediated through the regulation of biochemical, physiological, and molecular responses in plants (Wadas, 2022). Beyond biochemical processes and molecular expressions, Si also enhances plant resilience against biotic stresses by increasing cell wall strength and structural integrity, which increases resistance to insect pests, bacterial, and fungal diseases (Rodrigues et al., 2015). This reinforcement of the cell wall is achieved through the deposition of silica bodies, known as phytoliths or plant opal, beneath the cuticle and within the intercellular spaces of plant tissues (Heckman, 2013). Abiotic stresses such as extreme temperatures, UV radiation, and drought are mitigated through the promotion of longer roots and wider, thicker leaves, which improve water uptake and retention, thereby enhancing water use efficiency (WUE) (Epstein, 1999; Currie and Perry, 2007).

### 3.6 Quantitative evidence for Si-mediated drought tolerance across crops

The effects of Si on drought tolerance have been quantitatively assessed across various crops, consistently demonstrating benefits in biomass, water-use efficiency, and yield. In a multi-year field study, Si supplementation resulted in an increase of shoot mass in wheat by up to 73% under drought conditions and restored grain yield in drought-stressed plants to levels statistically comparable to well-watered, non-Si control plants. Leaf-level water-use efficiency increased by 32-74%, depending on the Si application rate, and tiller number rose by up to 36%, indicating both structural and physiological advantages (Johnson et al., 2022). Similarly, another field trial involving wheat found that the application of a moderate Si rate (20 kg ha^-^¹) during the stem elongation phase over two years led to a grain yield increase of 16% in the first year and 24% in the second year, relative to control plots experiencing water limitation (Saleh and Soliemanzadeh, 2026). In sorghum, a controlled drought experiment indicated that Si supplementation enhanced drought tolerance by 25.5% and boosted the grain yield of drought-stressed plants by 24% compared to untreated drought-stressed plants, while also improving height growth and total dry matter (Avila et al., 2020). Furthermore, a study comparing irrigated and water-deficit sorghum revealed that Si increased total dry weight and water-use efficiency by 30% and 36%, respectively, and improved osmotic and antioxidant traits associated with enhanced yield stability (Ahmed et al., 2011). Similar outcomes have been documented in Si-excluding crops. In soybean, a controlled experiment demonstrated that Si reduced malondialdehyde (MDA) and H_2_O_2_ by 42% and 12%, respectively, while increasing water-use efficiency by 25% compared to the control group (Abdullah et al., 2025). Likewise, in lentils, Si enhanced the photosynthetic rate by 1.2-2.3 times, which correlated with a yield increase of up to six times under controlled conditions and 2.9 times in field conditions in drought-susceptible genotypes (Biju et al., 2023b). In black gram, Si increased root length by 54%, thereby enhancing water potential by 12% under combined drought and salinity stress (Ahmad et al., 2024a).

Although variations exist across drought studies, both pot and field experiments demonstrate that Si application increases biomass by an average of 20% to 50%. This enhancement is accompanied by improved water status and consistent reductions in oxidative damage, all of which contribute to yield benefits ranging from 15% to 90% under both controlled and field conditions, depending on the crop species/genotype and the severity of the stress. While excluders typically accumulate lower levels of Si in their tissues, the relative improvements compared to their drought-stressed baselines are often significant. These quantitative gains are commonly associated with Si-induced enhancements in root length and surface area, improved root hydraulic conductance and aquaporin activity, regulated stomatal conductance, and strengthened antioxidant and osmotic adjustment activities, which collectively enable crops to sustain growth and reproductive success under reduced soil moisture (Wang et al., 2021a; Bardhan et al., 2024). These findings establish that Si is not limited to short-term pot experiments but is a validated, practical tool for enhancing crop drought resilience across multiple growing seasons.

## 4 Si-mediated drought tolerance mechanisms

**Figure 3:**
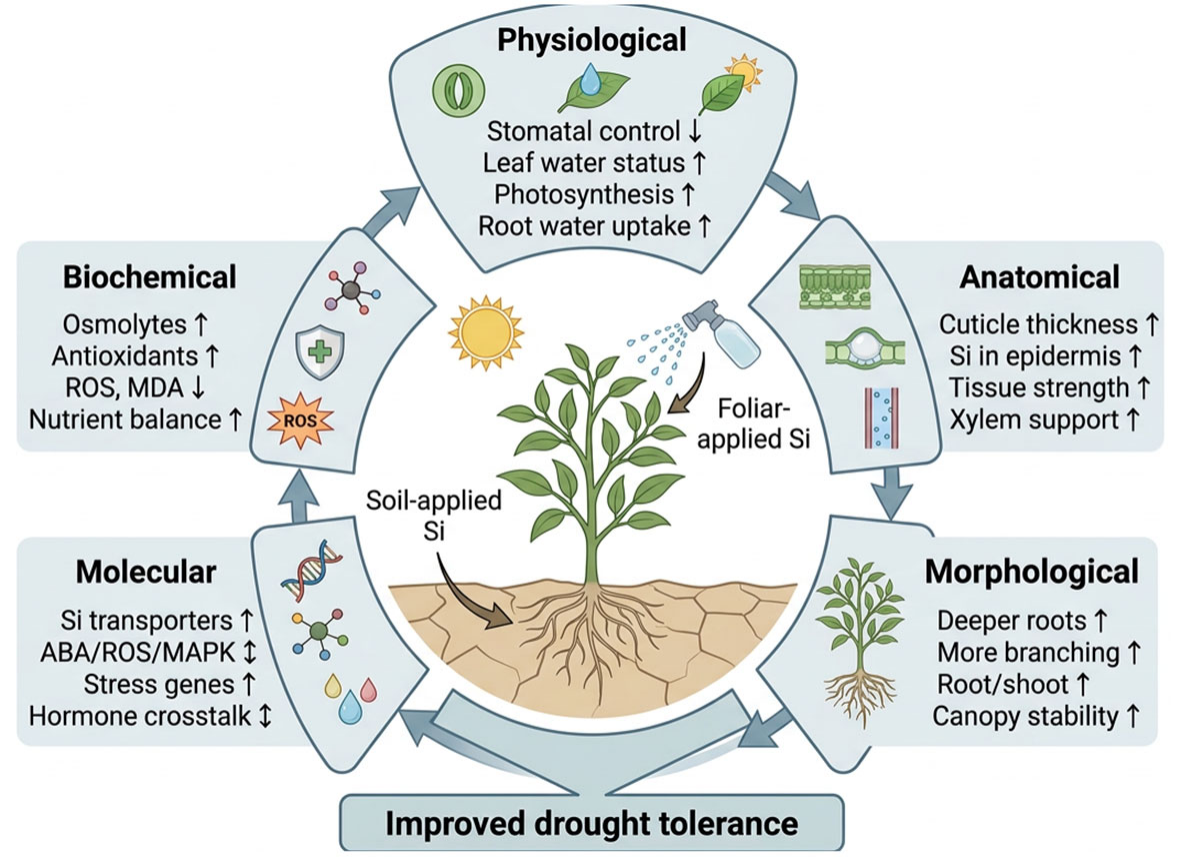
Silicon-mediated mechanisms for enhancing plant drought tolerance.

Recent interest in Si has increased significantly due to its potential to enhance plant resilience under stress conditions, by enhancing various plant processes as illustrated in **Figure 3**. These mechanisms result in the sustenance of drought avoidance and tolerance strategies.

Silicon facilitates several critical processes that collectively enhance plant water relations during drought conditions (Bhardwaj and Kapoor, 2021). These processes include promoting root growth, increasing water uptake, facilitating osmotic adjustment, and modulating aquaporin activity. The supplementation of Si increases the root surface area, thereby improving the plant’s capacity to access and absorb water from the soil, even under drought conditions (Wang et al., 2021b; Tripathi et al., 2021b) This is accompanied by the upregulation of root aquaporins, which are membrane proteins responsible for facilitating water movement, resulting in increased root hydraulic conductivity and more efficient water transport throughout the plant (Wang et al., 2021a). In addition to structural changes, Si contributes to the accumulation of osmolytes, such as soluble sugars and amino acids, by enhancing osmotic adjustment capacity and regulating related metabolic pathways (Biju et al., 2017). Thereby, increasing the osmotic driving force that helps maintain cell turgor and water content which contributes to improved stress tolerance in plants. Furthermore, Si stimulates antioxidant enzyme activity, which alleviates drought-induced oxidative stress in plant tissues and preserves cellular homeostasis for optimal water retention (Zhang et al., 2018; Saja-Garbarz et al., 2024). Notably, Si influences stomatal behaviour, aiding in the balance of water conservation with photosynthetic efficiency by modulating stomatal closure through hormonal regulation, primarily via abscisic acid (ABA) signalling (Maghsoudi et al., 2015; Patel et al., 2021). These integrated effects highlight the complexity of various mechanisms involved in Si-mediated plant water balance under drought conditions. **Table 2** presents a summary of several studies that report the drought-mitigating effects of Si on various plant species.

**Table 2:** Summary of Si’s role in enhancing drought tolerance in crops: physiological and biochemical effects of Si application under controlled and field conditions.

Crop	Environmental condition	Si Source	Dose/concentration	Application method	Results	References
Si accumulators						
Wheat	Field	Diatomaceous earth (Agrisilica)	39, 78 & 117kg Si ha^-1^	Soil amendment	Increased water use efficiency, biomass, shoot carbon content and grain yield	(Johnson et al., 2022)
Wheat	Field	Sodium Silicate	6mM	Foliar application	Increased photosynthetic pigments, chlorophyll stability index, membrane stability index and relative water content	(Maghsoudi et al., 2016b)
Sorghum	Controlled	Potassium silicate	5g per 15kg pot at sowing & 100ml Si solution at 100mg L^-1^ (3.5mM) per pot at 23DAS	Soil amendment	Increased plant dry weight, photosynthetic and transpiration rates	(Hattori et al., 2005)
Rice	Controlled	Potassium silicate	1.5mM	Soil amendment	Increased photochemical efficiency, and regulated absorption of K, Na, Ca, Mg, Fe	(Chen et al., 2011)
Sugar cane	Controlled	Potassium silicate	2.5mM	Fertigation and foliar application	Increased efficiency of PSII, antioxidant enzyme activities, and decreased electrolyte leakage	(Teixeira et al., 2022)
Si excluders						
Lentils	Controlled	Sodium metasilicate	2mM	Soil amendment	Increased germination percentage, increased antioxidant enzyme activities, decreased lipid peroxidation and regulated osmolyte concentration	(Biju et al., 2017)
Soybean	Controlled	Silicic acid	300ml Si solution at 200mg L^-1^ (7mM) per, 5.5L pot	Soil amendment	Increased biomass and regulated phytohormone levels	(Hamayun et al., 2010)
Squash	Field	Silicic acid	2g L^-1^ & 4g L^-1^	Foliar application	Increased vegetative growth, relative water content, antioxidant enzyme activities, increased fruit setting and fruit yield	(Salim et al., 2021)
Tomato	Controlled	Potassium silicate	2.5mM	Hydroponic nutrient solution	Increased crop water uptake through enhanced root hydraulic conductance	(Shi et al., 2016)
Peanut	Controlled	Sodium metasilicate	2mM	Hydroponic nutrient solution	Increased uptake and transport of mineral nutrients, regulated osmolyte concentration and phytohormone levels	(Patel et al., 2021)

### 4.1 Physiological mechanisms

#### 4.1.1 Improved photosynthesis

Photosynthesis is the fundamental process that generates energy and structural molecules for plant growth and maintenance (Knox et al., 2010). Consequently, it plays a crucial role in determining the level of biological productivity in plants and is significantly affected by environmental stressors. Under drought conditions, the concentrations of photosynthetic pigments, such as chlorophyll and carotenoids, are diminished due to the reduced capacity of plants to absorb moisture and nutrients, leading to nutrient deficiency symptoms manifested as decreased chlorophyll content (Yang et al., 2021). The reduction in chlorophyll concentration in stressed plants may also be attributed to enhanced chlorophyll degradation, resulting from increased chlorophyllase activity (Patel et al., 2021) reported that the Si-induced increase in chlorophyll content could be associated with the inhibition of chlorophyll degradation, potentially due to the regulation of chlorophyllase activity by Si. However, research indicates that the effect of Si on photosynthesis, as with other plant processes, is not confined to a singular mechanism but rather a combination of interconnected mechanisms. In drought-stressed sunflower plants, Gunes et al. (2008) observed that Si application enhanced photosynthetic pigments by promoting the uptake of macro- and micro-nutrients from the soil. This finding is substantiated by studies indicating that Si enhances water and nutrient uptake, consequently improving photosynthetic efficiency in drought-stressed plants (Chen et al., 2011). This is evidenced by increased leaf gas exchange, elevated levels of chlorophyll a and b, carotenoids, the chlorophyll stability index, and carboxylation efficiency (Maghsoudi et al., 2016b; Teixeira et al., 2022; Verma et al., 2022b, 2020). The cumulative effects of Si on various plant processes culminate in enhanced photosynthetic efficiency, which subsequently leads to increased growth under stress conditions.

#### 4.1.2 Regulation of stomata conductance and transpiration rate

During periods of drought, plants close stomata to minimise water loss through transpiration and conserve soil water. However, this process restricts the diffusion of CO_2_ into the leaves, thereby limiting photosynthesis (Varone et al., 2012). Silicon mitigates the effects of drought stress in plants by regulating stomatal conductance. In a field study on wheat, Si was found to enhance CO_2_ uptake in plants under both normal and drought-stressed conditions (Maghsoudi et al., 2016b). This was attributed to increased plant water content, which results in greater turgidity and higher stomatal conductance. Wang et al. (2021b) proposed that the increase in plant water content could also be attributed to Si’s facilitation of root water uptake, due to root growth and hydraulic conductivity. Chen et al. (2011) observed that Si improved root traits, including root length, root volume, root surface area, and root activity, thereby enhancing water and nutrient absorption in rice. This increased water uptake also increases transpiration (Chen et al., 2018). The synergistic effect of these mechanisms, in conjunction with osmotic adjustment that enables plants to retain more water under drought stress, leads to greater water-use which in turn sustains photosynthetic activity and plant growth.

An unresolved question pertains to whether the implementation of drought treatments in controlled environment experiments may exaggerate the benefits of Si-mediated drought tolerance. Many controlled drought studies aim to maintain a specific soil moisture level, necessitating frequent irrigation. Consequently, plants with higher transpiration rates receive more water. This experimental setup does not accurately replicate field drought conditions, where soil water is limited, and rapid early depletion could adversely affect long-term survival and reproductive growth. As noted by Puértolas et al. (2017), frequent irrigation to maintain field capacity may alter plant water relations in ways that do not accurately reflect real-world drought scenarios. This is because it can result in uneven soil moisture distribution, with water added to maintain a target weight being absorbed by the upper soil layers, leaving the top of the pot wet while the bottom dries out. Conversely, Moshelion et al. (2024) pointed out that completely withholding water might not subject plants to the same level of stress. This could have significant implications on the results, as those with greater biomass would transpire more, absorb more water, and thus experience severe stress more quickly and intensely than those with lower transpiration rates. The complex interactions between plant genotype and their environment present a significant experimental challenge that requires careful consideration in the experimental plan.

Research has demonstrated that Si facilitates root development, thereby enhancing the roots’ capacity for water absorption, which subsequently improves canopy physiology under drought conditions (Ashfaq et al., 2023). This may elucidate why certain field studies also report an increase in plant transpiration attributable to Si (Maghsoudi et al., 2016b). With improved canopy conditions, Si-treated plants are likely to exhibit increased transpiration and, consequently, a higher water demand, potentially supported by augmented root growth. However, in controlled settings if water is entirely withheld, Si-treated plants, which possess greater biomass due to enhanced canopy, may experience heightened stress, potentially influencing the outcomes. Further research employing diverse methods to induce drought could provide additional insights. Discrepancies have been observed in the effects of Si on transpiration under drought stress across various studies, as depicted in **Table 3**. Regardless of whether transpiration is increased or decreased, these studies consistently indicate that Si enhances water use efficiency (WUE). A detailed elucidation of the underlying biochemical and molecular mechanisms modulated by Si could further clarify the physiological basis of this improvement.

**Table 3:** Effects of drought and silicon application methods on transpiration in various crops.

Crop	Environmental condition	Drought application method	Si application method	Effects on transpiration	References
Wheat	Field	Total water withdrawal	Soil amendment	Decreased transpiration and increased WUE	(Johnson et al., 2022)
Wheat	Field	Total water withdrawal	Foliar application	Increased transpiration and WUE	(Maghsoudi et al., 2016b)
Sorghum	Controlled	Targeted moisture content	Soil amendment	Increased transpiration but no effect on WUE	(Hattori et al., 2005)
Rice	Controlled	Targeted moisture content	Soil amendment	Increased transpiration and WUE	(Chen et al., 2011)
Sugar cane	Controlled	Targeted moisture content	Fertigation and foliar application	Decreased transpiration and increased WUE	(Teixeira et al., 2022)
Sugar cane	Controlled	Targeted moisture content	Foliar application	Increased transpiration and WUE	(Verma et al., 2021b)
Tomatoes	Controlled	Targeted moisture content	Foliar application	Increased transpiration and WUE	(Morato de Moraes et al., 2020)

### 4.2 Biochemical processes

#### 4.2.1 Promotion of antioxidant enzyme activities

Silicon enhances the activities of antioxidant enzymes in plants under drought stress. Drought stress increases the production of ROS in plants, resulting in imbalanced oxidative homeostasis and consequently, oxidative damage in plant cells (Yang et al., 2021). Reactive oxygen species, such as superoxide anion (O_2_^-^), hydrogen peroxide (H_2_O_2_), hydroxyl radical (OH) and singlet oxygen (^1^O_2_), are chemically active metabolites produced from the incomplete reduction of oxygen during plant metabolic processes. Under stress conditions, their production is elevated, resulting in detrimental effects such as lipid peroxidation, protein denaturation, and DNA strand breakage (Kapoor et al., 2015). Reactive oxygen species are produced through various metabolic pathways in plants, including the chloroplast, mitochondria, and plant plasma membrane, as well as other locations. The chloroplast serves as the primary source (Dietz, 2016). Light energy absorption decreases in drought-stressed plants, with reduced NADP^+^ supply due to impaired CO_2_ fixation. This condition increases photosynthetic electron transfer, where molecular oxygen (O_2_) acts as an electron acceptor, resulting in the formation of superoxide anion (O_2_^-^), which elevates ROS levels in plants (Pospíšil, 2012). In mitochondria, superoxide is generated through the interaction of electrons leaked during electron transfer in the respiratory chain, with O_2_ (Shields et al., 2021).

Silicon enhances the activity of enzymatic antioxidants, including superoxide dismutase (SOD), peroxidase (POD), catalase (CAT), and ascorbate peroxidase (APX), which function to scavenge ROS and maintain oxidative homeostasis (Bhardwaj and Kapoor, 2021; Gong et al., 2008). This enhancement increases the enzymes’ capacity to manage elevated ROS levels induced by stress, thereby mitigating oxidative damage. Additionally, Si modulates antioxidant defences in response to drought at both biochemical and transcriptional levels, resulting in more effective detoxification of ROS (Ma et al., 2016). Plants treated with Si typically exhibit increased activities of key antioxidant enzymes, which facilitate the conversion of superoxide and hydrogen peroxide into less harmful products, ultimately reducing lipid peroxidation and membrane damage (Biju et al., 2021; Teixeira et al., 2022). These alterations in enzyme activity are often influenced by the upregulation of genes encoding antioxidant enzymes and components of the ascorbate–glutathione cycle (Ma et al., 2016). This observation suggests that Si influences redox-sensitive signalling pathways and transcription factors that regulate ROS-scavenging networks. Mechanistically, Si may stabilise chloroplast and mitochondrial membranes, improve water and nutrient status, and modulate abscisic acid (ABA)/ROS/mitogen−activated protein kinase (MAPK) signalling, which aids in reducing excessive ROS generation while enhancing the plant’s capacity to neutralise ROS (Mostofa et al., 2021).

Superoxide dismutase is a critical metalloenzyme that constitutes the first line of defence within the protective enzyme system by converting O_2_^-^ into H_2_O_2_. While CAT and POD are responsible for the decomposition of H_2_O_2_ into H_2_O and O_2_ (Yang et al., 2021). These processes contribute to the maintenance of oxidative homeostasis and the protection of cellular structures (Verma et al., 2021b, 2020; Wang et al., 2021b). This is evidenced by the reduction of oxidative stress markers such as malondialdehyde (MDA) and hydrogen peroxide (H_2_O_2_) (Abdullah et al., 2025). El-Okkiah et al. (2022) found that Si increased the activities of POD and CAT while reducing MDA content in rice. Comparable findings have been reported in soybean (Shen et al., 2010), lentils (Biju et al., 2017), sugarcane (Teixeira et al., 2022), and wheat (Ashfaq et al., 2022). Silicon also increases the synthesis of non-enzymatic antioxidants such as glutathione and ascorbate which are plants’ second line of defence against ROS (Kim et al., 2017; Bhardwaj and Kapoor, 2021). Collectively, this Si-mediated reprogramming of enzymatic and non-enzymatic antioxidant defence system, enables plants to maintain cellular redox homeostasis and protect photosynthetic and membrane integrity during drought stress, significantly contributing to enhanced drought tolerance (Kapoor et al., 2015; Teixeira et al., 2022).

#### 4.2.2 Osmotic adjustment

Osmotic adjustment protects plant cellular membranes and proteins while maintaining water balance under drought stress conditions (Zhang et al., 2018). This maintains cellular homeostasis in response to osmotic stress by accumulating solutes in cells to lower the osmotic potential, thereby increasing water uptake and retention. Osmotic adjustment is associated with increased synthesis of both enzymatic and non-enzymatic antioxidants, as well as organic osmolytes, including proline, glycine betaine, polyamines, soluble sugars, and free amino acids (Arif et al., 2021; Hassan et al., 2022). These osmolytes accumulate during stress conditions in plants to regulate internal physiological processes and promote growth and development (Seleiman et al., 2021). Under drought conditions, elevated levels of osmolytes such as proline enhance water affinity in plants, thereby improving their water retention efficiency (Patel et al., 2021). Proline also mitigates osmotic stress by scavenging hydroxyl radicals from plant cells (Arif et al., 2021). Elevated proline levels in plants are often indicative of increased stress. In instances of prolonged stress, plants may accumulate proline to toxic levels, potentially resulting in stress-related damage (Pei et al., 2010). However, Si regulates proline levels and prevents osmolyte toxicity (Pei et al., 2010; Biju et al., 2021). The regulatory mechanism operates by integrating Si with stress signalling pathways, thereby influencing proline metabolism through its impact on stress hormone signalling, particularly abscisic acid (ABA), and antioxidant systems (Ali et al., 2018; Al-Huqail et al., 2019). Additionally, Si interacts with other osmolytes, such as glycine betaine and total soluble sugars, to sustain optimal osmotic potential without inducing cellular toxicity (Abdullah et al., 2025). This intricate regulation ensures that osmolyte concentrations are maintained within optimal ranges, adequate for osmotic adjustment and stress protection, yet not so excessive as to disrupt cellular metabolism or cause osmolyte toxicity. Consequently, this regulation enhances membrane stability (Biju et al., 2021), thereby enabling cells to sustain elevated turgor pressure and stabilising cellular and membrane structures. Some documented effects of Si on osmolytes in plants under drought stress include a reduction in proline accumulation in maize (Kaya et al., 2006), a decrease in proline and glycine betaine concentrations in lentils (Biju et al., 2021), and an improvement in production of total phenolics and total soluble protein in soybean (Abdullah et al., 2025).

#### 4.2.3 Regulation of phytohormones

Plant hormones ABA, salicylic acid (SA) and Jasmonic acid (JA) serve as chemical signal molecules that regulate growth, development, and stress responses in plants (Nadarajah, 2020; Tayyab et al., 2020; Fadiji et al., 2022). These hormones control antioxidant systems and facilitate osmotic adjustments, thereby reducing reactive oxygen species (ROS), malondialdehyde (MDA), and electrolyte leakage (Arif et al., 2021). ABA is a crucial hormone in plant physiology, essential for imparting tolerance to various abiotic stresses. Under drought stress, ABA activates drought-responsive genes and coordinates complex cell signalling pathways, initiating enhanced resistance responses such as stomatal closure and modifications to the root system. Silicon modulates plant hormonal networks during drought conditions by coordinating hormone biosynthesis, degradation, signalling, and downstream transcription factors (Malik et al., 2021; Tripathi et al., 2021a). Instead of merely elevating ABA levels in response to water deficit, Si regulates ABA homeostasis. For example, in rice, Si modified the expression of genes associated with ABA biosynthesis and degradation, leading to a moderated accumulation of ABA that is adequate for stomatal regulation without inducing excessive growth inhibition (Kim et al., 2014 While in sorghum, it decreased 1-aminocyclopropane-1-carboxylic acid (ACC), and increased polyamine synthesis, adjusting the balance between defence and growth (Yin et al., 2014).

Research indicates that Si influences both ABA-dependent and ABA-independent pathways by modulating key transcription factors that regulate a spectrum of stress-responsive genes involved in osmotic balance, ROS detoxification, and cell wall restructuring remodelling (Wang et al., 2021b). Both the Si transporter (Lsi1) and Si-regulated genes such as OsNAC5 and OsDREB2A are suggested to contain cis-elements that respond to ABA, JA, SA, and other phytohormones (Gómez-Merino et al., 2020). This evidence supports the theory that Si functions either upstream or alongside hormonal signalling modules to adjust hormonal interactions during drought conditions (Tripathi et al., 2021a). Through its multifaceted influence on hormone biosynthesis, receptor and kinase cascades, including ABA- linked MAPK pathways and transcriptional networks, Si helps plants attain a more adaptable hormonal state, thereby maintaining stomatal regulation and root development while mitigating stress-related damage during drought (Mostofa et al., 2021).

Silicon supplementation has been demonstrated to enhance the biosynthesis of growth-related hormones such as auxin, zeatin, and gibberellin, while concurrently inhibiting the synthesis and accumulation of ABA, thereby preventing stomatal closure during drought conditions (Gao et al., 2022). Si influences the signal transduction pathways of auxin, cytokinin, gibberellin, and salicylic acid, and modulates the expression of genes involved in hormone pathways, thereby enhancing stress-responsive hormonal cross-talk for improved growth and drought adaptation (Patel et al., 2021; Tripathi et al., 2021a). It also facilitates root development by enhancing auxin and cytokinin signalling, which promotes lateral root formation and increases water uptake capacity (Gao et al., 2022). The impact of Si on phytohormones under drought stress is multifaceted, promoting the biosynthesis of growth hormones, suppressing ABA-induced stomatal closure, and regulating multiple hormone signal transduction pathways to collectively enhance the drought resilience of plants. This modulation restores hormone ratios that favour stomatal opening, improved photosynthetic rates, and enhanced water use under limited water conditions (Gao et al., 2022). Additionally, Si supports antioxidant defence through interconnected changes in hormone biosynthetic and metabolic processes, ROS scavenging, and membrane stability.

## 5 Morpho-anatomical modifications

Water stress induces morphological alterations due to changes in cell expansion patterns. Cell expansion is a fundamental process in which cells increase in size and is essential for development of cellular structure and tissue growth and development (Gall et al., 2015). This process is contingent upon turgor pressure surpassing the cell wall yield threshold. Water stress impedes cell expansion by causing turgor pressure to fall below the critical yield threshold, thereby affecting plant structure and overall growth. Silicon induces morpho-anatomical modifications in plants subjected to drought stress (Malik et al., 2021). Silicon deposition fortifies cell walls through the formation of covalent bonds with their constituent components. This reinforcement not only enhances the mechanical strength of the cell walls but also maintains the requisite flexibility necessary for cellular expansion and contraction during osmotic adjustments (Singh et al., 2023). Silicon-induced alterations include improvements in root growth, root driving force, root hydraulic conductivity, and root water uptake (Wang et al., 2021a). Enhanced silicification in shoots and the endodermal cells of sorghum roots was associated with increased drought resistance (Lux et al., 2002). The promotion of suberin and lignin deposition, along with the enhancement of endodermal and exothermal casparian band formation, reduced radial oxygen loss in the roots of rice treated with Si (Fleck et al., 2011). Furthermore, improvements were observed in the structures of mesophyll cells, chloroplasts, and mitochondria in the leaves of rice seedlings exposed to acid rain when treated with Si. This treatment helps preserve the structure and functionality of stomatal cells by reducing the influx of acid rain into the plant (Ju et al., 2017). These Si-induced modifications are achieved through coordinated changes at the cellular, tissue, and organ levels. This includes the synergistic effects of Si-induced morphological alterations alongside physiological adaptations, such as increased leaf thickness and enhanced cuticle development, which collectively contribute to the maintenance of elevated relative water content and photosynthetic efficiency (Mastalerczuk et al., 2025). Zargar et al. (2019) proposed that the genetic modification of root characteristics in Si-excluding crops could potentially enhance their Si uptake capabilities.

## 6 Molecular mechanisms

Silicon-modulated drought tolerance relies on a complex network of interconnected molecular processes, encompassing hormonal signalling, transcriptional networks, and downstream stress-response genes (Manivannan and Ahn, 2017). These mechanisms collaboratively facilitate the alteration of plant perception, signalling, and response to water deficits. At the hormonal level, Si influences the transcription of ABA biosynthetic and catabolic genes, including those analogous to NCED. This modulation regulates ABA accumulation during periods of drought, facilitating the efficient regulation of stomata while preventing excessively high ABA levels, thereby averting prolonged growth arrest (Malik et al., 2021). Si also influences JA, SA, and ethylene (ET) signalling. Si treatment modifies expression of JA- and ET-related biosynthetic and signalling genes, shifting the balance between defence and growth and feeding into ABA−dependent and ABA−independent drought pathways (Kim et al., 2016; Wang et al., 2021). Transcriptome studies indicate that Si upregulates important transcription factors, including members of the NAC and DREB families. These transcription factors subsequently activate a range of drought-responsive genes that encode late embryogenesis abundant (LEA) proteins, enzymes for osmolyte biosynthesis, components for ROS scavenging, and cell−wall−modifying enzymes (Jiang et al., 2022).

Silicon modulates the expression of downstream stress response genes associated with redox and osmotic homeostasis. Silicon has been shown to upregulate transcripts encoding reactive ROS-scavenging enzymes, including SOD, CAT, APX, various class III peroxidases, and enzymes involved in the ascorbate–glutathione cycle. Concurrently, Si reduces the expression of NADPH oxidases in specific contexts. Collectively, these molecular modifications contribute to the modulation of ROS production and enhance detoxification capacity under drought conditions (Ma et al., 2016; Ning et al., 2026). Silicon-treated plants exhibit elevated expression of genes associated with the biosynthesis of compatible solutes, including proline, glycine betaine, and soluble sugars. This encompasses choline monooxygenase (CMO) and other enzymes related to osmolytes, which contribute to osmotic adjustment and the maintenance of turgor pressure (Manivannan and Ahn, 2017). RNA sequencing of Si-treated drought-stressed lentil plants demonstrated an upregulation of genes related to photosynthesis, osmoregulation, antioxidant systems, and signal transduction (Biju et al., 2023). Although this species is classified as a silicon excluder, as indicated in **Table 1**, it still exhibited molecular-level enhancements in response to silicon application. This finding supports the hypothesis that silicon excluders can also derive benefits from the effects of silicon, as evidenced in previous studies on silicon excluders, including soybean (Deshmukh et al., 2013; Devkar et al., 2025).

Silicon influences the genes involved in cell wall and membrane remodelling by upregulating expansins, cellulose synthases, and lignin-biosynthetic enzymes. This process reinforces cell walls, modifies elasticity, and regulates aquaporin genes from the PIP and TIP families, which control membrane water transport and root hydraulic conductivity (Chen et al., 2018; Tripathi et al., 2021a). However, similar to the diverse effects of Si in different plant species, its influence on gene expression also exhibits variability. Studies indicate that the expression level of the Si-transporting aquaporin gene Lsi1, which is constitutively expressed in the roots of rice plants, is upregulated by Si supplementation (Tripathi et al., 2021a). In barley, while studies demonstrate a significant increase in Si uptake due to the presence of the HvLsi1 Si influx transporter in the basal root, the expression level of the HvLsi1 gene remains unaffected by Si supplementation (Chiba et al., 2009). In soybean, although the expression of GmNIP2–2 was found to significantly increase Si absorption and the expression of the Si efflux transporter GmHiSil2c was significantly elevated in the leaf following silicon treatment, qRT-PCR analysis of the plant roots revealed a significant decrease in the expression levels of Si influx transporters Lsi homologs GmNIP2–1 and GmNIP2–2 in Si treated plants compared to the control (Deshmukh et al., 2013; Devkar et al., 2025). The reduced expression of the Lsi1 influx transporters GmNIP2‐1 and GmNIP2‐2 in response to Si treatment suggests the existence of a potential homeostatic feedback mechanism. This results in the downregulation of influx transporters to prevent overaccumulation, similar to the observations made in rice (Mitani-Ueno et al., 2016).

The Si-induced transcriptional landscape facilitates a more rapid transition of plants from growth to protective modes in response to water deficits, enabling a return to growth once the stress is mitigated. This dynamic results in a more resilient drought response, characterised by reduced cellular damage and enhanced yield stability. Collectively, these gene-level modifications illustrate the capacity of Si to reprogram diverse molecular pathways, thereby establishing a drought-primed state that enables plants to maintain water status, safeguard cellular structures, and sustain metabolic functions under conditions of stress.

## 7 Conclusion and future directions

This review synthesises current evidence regarding the mechanisms underlying Si-mediated drought tolerance in plants. It emphasises that Si enhances plant water relations, modulates stomatal conductance and transpiration, facilitates osmotic adjustment, induces morpho-anatomical changes, regulates phytohormone signalling, enhances antioxidant defence, supports photosynthetic performance, and influences gene expression, all of which contribute to increased drought resilience. While some discrepancies exist concerning Si’s effects on transpiration, the literature generally concurs that Si improves water-use efficiency and confers drought tolerance, irrespective of whether transpiration rates increase or decrease. To address these inconsistencies, future research should employ diverse and well-defined drought-induction protocols while meticulously controlling for experimental factors. This approach will facilitate a more accurate attribution of the observed effects specifically to Si. There should also be a concerted effort to understand Si-mediated responses in Si-excluding species, particularly at the molecular and cellular levels, to determine whether these species utilise distinct signalling and regulatory networks compared to Si accumulators. Furthermore, more field studies are required to bridge the gap between controlled and field experiment findings and address the role of Si in modulating plant-microbiome interactions under drought stress. Such research will more effectively capture the complexities of agroecosystems and refine evidence-based recommendations for Si application in drought-prone production systems. Finally, optimising Si sources, doses, application timing, methods across various crop species, soil types, and climatic conditions will be crucial for translating mechanistic insights into practical strategies that enables the development of climate-resilient cropping systems.
