# Supplementary material for: Silicon-mediated drought resilience mechanisms in crops: from physiology to molecular insights
Source: Front Plant Sci. 2026 Apr 28;17:1788106. doi: 10.3389/fpls.2026.1788106 (PMC13162273; doi:10.3389/fpls.2026.1788106)
Supplement: Supplementary file 1 [file DataSheet1.pdf]

1. *Literature Search and Selection Methodology*

Although this is a narrative review, to ascertain the current state of primary, peer-reviewed research on the impact of exogenous Si application on drought stress mitigation in plants as of 2023, the literature search was conducted systematically to ensure relevant data from 1990 to 2023 (when the review started) is acquired. This search encompassed databases such as PubMed, Scopus, and Web of Science, using the following search terms: (((drought OR "drought stress" OR "water stress" OR "water deficit" OR "drought mitigation" OR "drought tolerance") AND ("climate change" OR "environmental stress" OR "abiotic stress")) AND (silicon OR "silicon fertiliser" OR "silicon fertilizer" OR "silicon application")) AND (plants OR "plant growth" OR "plant development" OR "crops" OR "crop production" OR "crop yield" OR yield). Additionally, supplementary studies were sourced from Google Scholar. Citation searching was also employed, particularly during the review and editing phases, at which point more recent studies were incorporated to expand the exploration of contemporary relevant literature. Furthermore, to maintain currency, searches were regularly updated through email alerts. This multifaceted approach ensured a comprehensive and up-to-date synthesis of pertinent information. The comprehensive search yielded 979 potential publications. These publications were systematically organised using Zotero referencing software, with meticulous removal of 264 duplicated records.

All articles underwent various screening stages, as depicted in the flow diagram (Figure 1). The initial screening was conducted based on the titles and abstracts to assess their relevance, resulting in the exclusion of 547 articles that involved interventions unrelated to the impact of drought stress and silicon Si-mediated drought stress tolerance in plants. Nine articles were inaccessible and thus could not be retrieved. A full-text examination of the remaining articles was subsequently performed, leading to the exclusion of articles for the following reasons:

1. Studies with that do not contribute to the understanding of the mechanisms involved in Si-induced drought stress tolerance.
2. Articles with methodological limitations or potential sources of bias, to ensure that the selected studies provide consistent, transparent and credible information.

This process resulted in the exclusion of an additional 77 articles, with 153 articles ultimately included in this review.

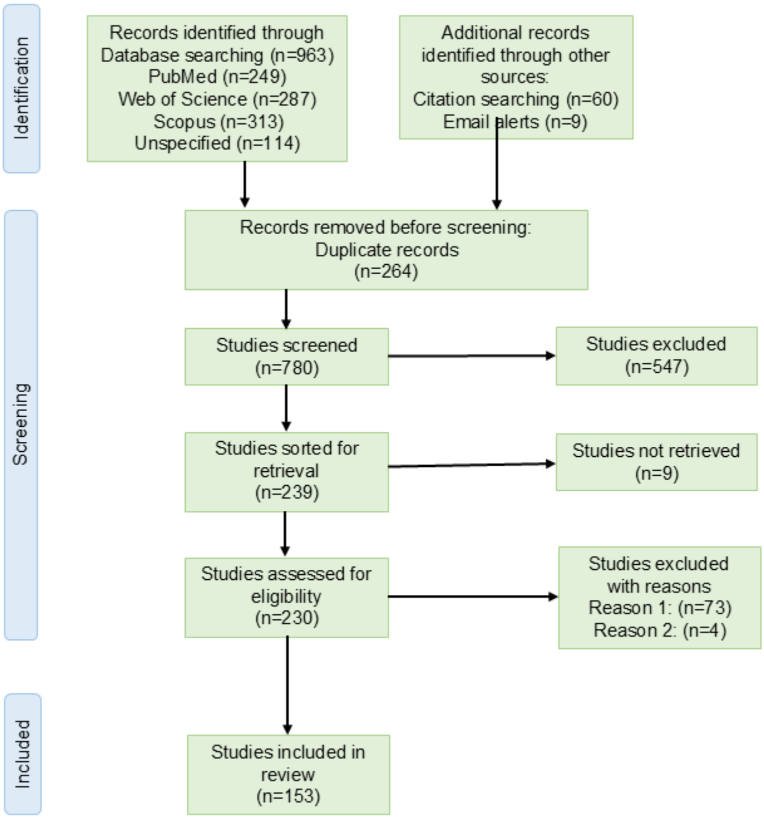

Figure 1: A flow diagram illustrating the selection process and workflow employed in determining the studies included in this review.

1.1. Temporal and thematic analysis of published literature on silicon's role in crop stress management included in this review

The studies encompassed in this review include both experimental research and review articles published between 1990 and February 2025. A significant portion of these studies were published in 2021 (17.5%) and 2022 (11.7%) (Figure 2a). Approximately 70% of the studies have been published since 2010, with a notable increase from 2020, accounting for 37% of the total publications. This trend indicates a growing interest in the application of Si for stress alleviation and enhanced crop production. Among the various journals, Frontiers in Plant Science published the highest number of articles (7.5%), followed by Plant Physiology and Biochemistry Journal (6.7%), and Plants (5.8%) (Figure 2b). Regarding the subject matter, the majority of studies focused on Si (34.2%), followed by research on Si application under drought stress (25.8%). Studies focusing solely on drought stress constituted 20.8%, while 15% explored Si for mitigating other environmental stresses, and 4.2% addressed the effects of Si on combined stresses (Figure 2c). All articles included in this review were either published in English or had an English manuscript, and they were distributed across a total of 73 different peer-reviewed journals and 4 government webpages.

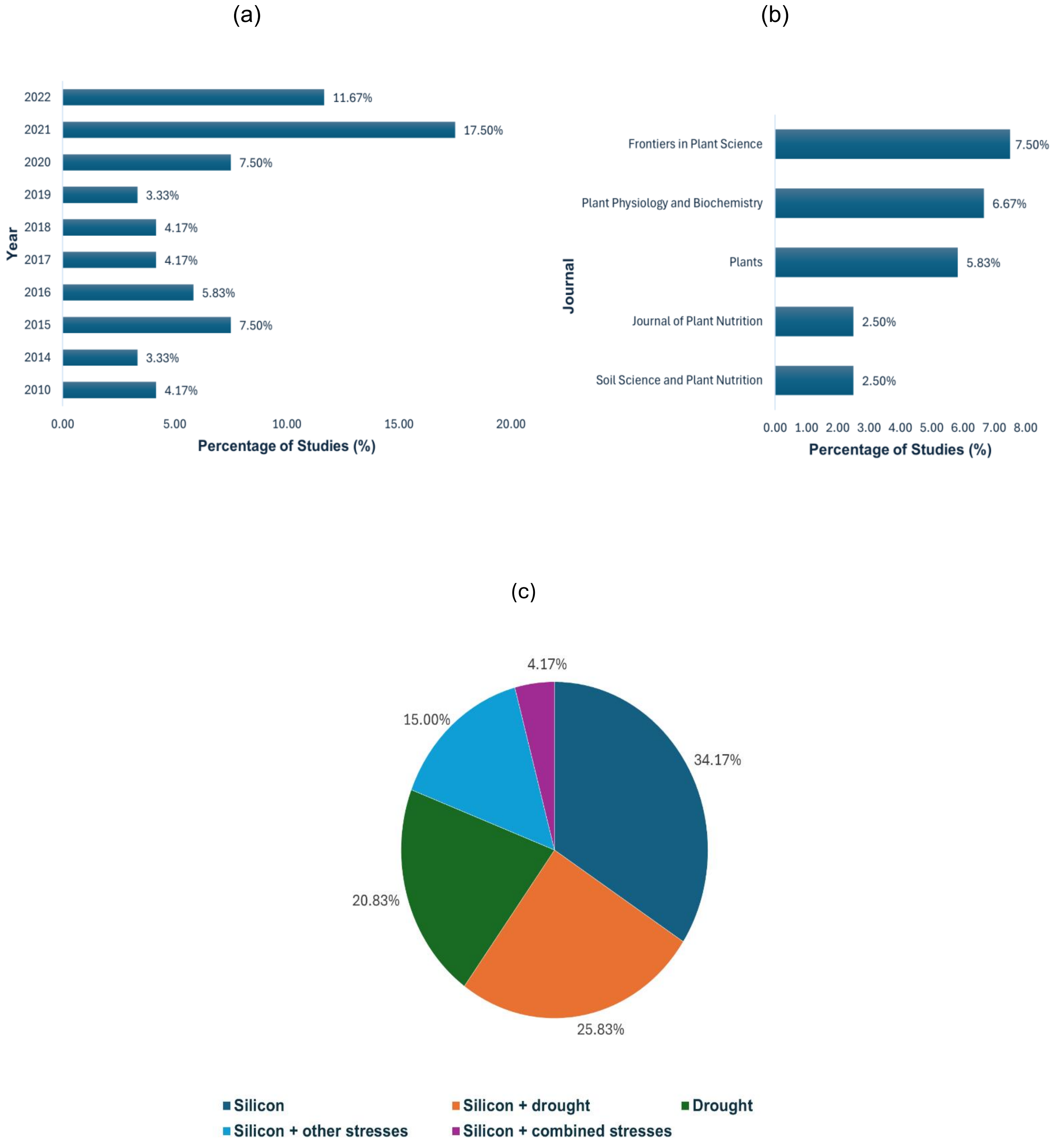

Figure 2: (a) Top 10 years with the highest percentage of included studies (b) Top 5 journals with the highest percentage of included studies (c) Proportion of studies investigating silicon, drought, other stresses and their combined impact
